# *Trichoderma*: The Current Status of Its Application in Agriculture for the Biocontrol of Fungal Phytopathogens and Stimulation of Plant Growth

**DOI:** 10.3390/ijms23042329

**Published:** 2022-02-19

**Authors:** Renata Tyśkiewicz, Artur Nowak, Ewa Ozimek, Jolanta Jaroszuk-Ściseł

**Affiliations:** 1 Analytical Laboratory, Łukasiewicz Research Network–New Chemical Syntheses Institute, Aleja Tysiąclecia Państwa Polskiego 13a, 24-110 Puławy, Poland; 2 Department of Industrial and Environmental Microbiology, Faculty of Biology and Biotechnology, Institute of Biological Science, Maria-Curie Skłodowska University, Akademicka 19, 20-033 Lublin, Poland; ewa.ozimek@mail.umcs.pl (E.O.); jolanta.jaroszuk-scisel@mail.umcs.pl (J.J.-Ś.)

**Keywords:** biological control, phytohormones, mycoparasitism, competition, antimicrobial metabolites, plant resistance induction

## Abstract

Rhizosphere filamentous fungi of the genus *Trichoderma*, a dominant component of various soil ecosystem mycobiomes, are characterized by the ability to colonize plant roots. Detailed knowledge of the properties of *Trichoderma*, including metabolic activity and the type of interaction with plants and other microorganisms, can ensure its effective use in agriculture. The growing interest in the application of *Trichoderma* results from their direct and indirect biocontrol potential against a wide range of soil phytopathogens. They act through various complex mechanisms, such as mycoparasitism, the degradation of pathogen cell walls, competition for nutrients and space, and induction of plant resistance. With the constant exposure of plants to a variety of pathogens, especially filamentous fungi, and the increased resistance of pathogens to chemical pesticides, the main challenge is to develop biological protection alternatives. Among non-pathogenic microorganisms, *Trichoderma* seems to be the best candidate for use in green technologies due to its wide biofertilization and biostimulatory potential. Most of the species from the genus *Trichoderma* belong to the plant growth-promoting fungi that produce phytohormones and the 1-aminocyclopropane-1-carboxylate (ACC) deaminase enzyme. In the present review, the current status of *Trichoderma* is gathered, which is especially relevant in plant growth stimulation and the biocontrol of fungal phytopathogens.

## 1. Introduction

Plant diseases play a direct role in destroying natural resources in agriculture and are said to be a major cause of reducing the annual level of food production in the world, which, depending on the source, is estimated at the level of 10–40% [1,2,3,4]. The most serious losses, both for natural and production ecosystems, are attributed to soil-borne pathogens, among which fungi constitute the most numerous group of plant disease agents, destroying as much as one-third of all crops annually [5]. According to the Food and Agriculture Organization of the United Nations (FAO), plant mycoses most often affect the five most important world crops—rice, wheat, corn, potatoes, and soybean [5]. Recently, more than 19,000 species of fungi that cause diseases of crops are known worldwide [6]. Most of the fungal phytopathogens belong to the Ascomycota and Basidiomycota phyla, the most serious of which are representatives of the genera *Cladosporium*, *Botrytis*, *Alternaria*, *Aspergillus*, *Verticillium*, *Pythium*, *Fusarium* (Ascomycota), and *Rhizoctonia* (Basidiomycota) [7,8,9,10,11,12,13].

In recent decades, the agricultural environment has been under severe pressure from chemical pesticides, the most popular method of protecting plants against fungal pathogens [4]. Though the high effectiveness of chemical plant protection products, there are concerns about their safe use and impact on the environment, as well as human and animal health [4]. The result of the abuse of chemical pesticides is an increase in the resistance of pathogens to pesticides, and the contamination of soil and ground waters. Furthermore, pesticides have a detrimental effect on non-target organisms (e.g., beneficial insects, including pollinators), soil microbiomes, and the general condition of terrestrial and aquatic ecosystems [14,15].

To protect the environment from the negative effects of chemical fungicides, various actions and strategies of sustainable food production systems are taken, including Integrated Pest Management (IPM) and organic farming [16,17]. One of these strategies is the use of Biological Control Agents (BCAs), based on living microorganisms or their metabolites, and products of natural origin that control the population of plant pathogens [16,18]. Over the last several decades, the most effort has been made to examine the effectiveness and practicality of non-pathogenic bacteria and fungi in the hope of commercializing them as BCAs [19,20]. As a result of the conducted studies, a large number of bacterial and fungal strains have been employed as BCAs, including *Pseudomonas* spp., *Bacillus* spp., *Streptomyces* spp., *Trichoderma* spp., *Glomus mosseae*, *Gliocladium virens*, *Pythium oligandrum*, and *Beauveria bassiana*, which successfully control the soil-borne diseases of valuable crops caused by fungi, oomycetes, bacteria, and nematodes [19,20,21]. The introduction of microbiological technologies to plant cultivation, based on microorganisms with biostimulating properties, is a subsequent element of a sustainable agricultural tactic [22]. The above solutions are in line with the current trends included in the strategic programs of the EU and are consistent with the assumption of the European Green Deal (EGD) and the EU Biodiversity Strategy for 2030, which emphasize the importance and necessity of agroecology development, agricultural biologicalization, and area increase in the plants grown in ecological production systems [23].

Among filamentous fungi, most of the BCAs belong to the phylum Ascomycota and are mainly representatives of numerous species belonging to the genus *Trichoderma* [24]. Numerous studies documented the beneficial properties of avirulent *Trichoderma* strains which allow their use in plant protection, biostimulation, and biofertilization [4,25,26]. The effectiveness of using *Trichoderma* in agriculture depends on their metabolic activity and the type of interaction with plants and other microorganisms. These fungi effectively colonize the rhizoplane, rhizosphere, and plant roots, and produce several metabolites with anti-microbial (cell wall degrading enzymes, antibiotics, volatile, and non-volatile compounds) and biostimulating (phytohormones, phytoregulators) features. Moreover, *Trichoderma* is known for its intensive absorption of root schedules and interaction not only with pathogenic microorganisms, but also interactions with the entire soil microbiome [24,26]. The present review focuses on the properties of *Trichoderma* to select the strains with the best parameters and predispose them to commercialization as a protective agent against fungal phytopathogens and biostimulators in specific plant crops.

## 2. Characteristics of the *Trichoderma* Allowing Its Use in Agriculture

*Trichoderma* is a genus of mostly asexual (the teleomorphic forms are *Hypocrea*) filamentous fungi, widespread around the world, usually colonizing rotting wood and other forms of organic plant matter [27]. *Trichoderma* is a dominant component of the mycobiome of various soil ecosystems (such as farmland, prairie, forests, salt marshes, and deserts) in all climatic zones, including temperate and tropical regions, Antarctica, and the tundra [4,28]. The *Trichoderma* genus is classified as cosmopolitan, saprotrophic fungi, often living as endophytes of woody plants [28]. The systematics and taxonomy of these fungi have evolved since 1794 when Persoon first introduced the generic name *Trichoderma* [29]. However, *Trichoderma* taxonomy has undergone a remarkable transformation since 1969, when Rifai concluded that the genus encompassed more than a few species and divided the strains studied into nine “aggregate” species based on their morphological features [26,30]. It is worth emphasizing that the taxonomy of the genus *Trichoderma* is complicated and belongs to the most dynamically developing branches of mycology [26,31]. According to the current MycoBank classification, the *Trichoderma* genus belongs to the domain Eukaryota, kingdom Fungi, division Ascomycota, subdivision Pezizomycotina, class Sordariomycetes, order Hypocreales, and family Hypocreaceae. The genus *Hypocrea*/*Trichoderma* already includes more than 300 molecularly and morphologically characterized species, many of which have not yet been formally described [32,33,34].

**Figure 1:**
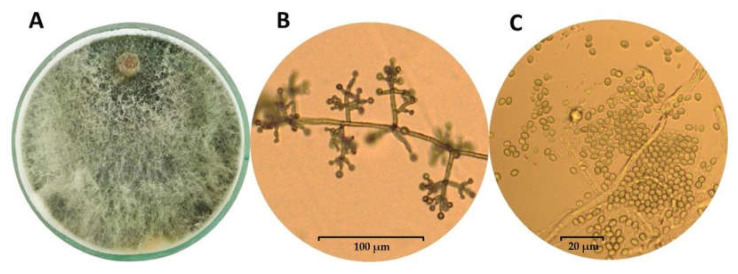
Growth of the mycelium of *Trichoderma koningiopsis* TkZ3A0 strain (**A**) and the appearance of conidiophores (**B**) and conidia (**C**) observed in microcultures on PDA medium using an Olympus BX53 Upright Microscope equipped with a Olympus XC30 camera.

The success of species belonging to the genus *Trichoderma* as biocontrol agents in the soil ecosystems results from their ability to rapid growth, the possibility of utilizing a variety of substrates, and resistance to many toxic chemicals, including fungicides (e.g., azoxystrobin, 3,4-dichloroaniline, and trifloxystrobin), herbicides, and other organic pollutants [28,35,36,37,38]. Other than that, *Trichoderma* was found to degrade some toxic contaminants through enzymes involved in cellulose/lignin degradation that have been shown to have xenobiotic-metabolizing enzyme potential [36,38]. For example, *T. viride* has been observed to degrade trinitrotoluene (TNT) [39], and *T. inhamatum* reduces hexavalent chromium [40]. Moreover, *Trichoderma* can adapt to changes in environmental conditions and abundantly produce conidia and chlamydospores [29,41,42]. The latest research indicates a much wider temperature range for the growth and development of *Trichoderma* than previously assumed and, above all, the optimal temperature for the production of metabolites important for interactions with the plant. Although the saprotrophic activity of *Trichoderma* strains is the highest in the range of 15 to 21 °C [43,44], it has been demonstrated for the DEMTkZ3A0 strain [26] that the temperature of 12 °C may be optimal for auxin and gibberellin synthesis and is suitable for high ACC deaminase activity. *Trichoderma* grows as a white or transparent (hyaline) mycelium on Potato Dextrose Agar (PDA), and the colony takes on various colorations (usually green-yellow or reddish) depending on sporulation color (Figure 1A) [41]. These fungi are characterized by multi-branched conidiophores with a pyramidal appearance and clusters of divergent, usually asymmetrical bent, flask-shaped/cylindrical phialides (Figure 1B), while the conidia are colorless or assume various shades of green, gray, or brown (Figure 1C) [29,35,41,45]. Moreover, some species produce a characteristic sweet or coconut odor [46]. *Trichoderma* is not only ubiquitous in the environment and is easy to be isolated, but can also be easily multiplied under controlled conditions on a variety of substrates and can be stored for many months without losing its viability and properties.

The potential of *Trichoderma* species as biological plant protection agents was first described in the early 1930s. Researcher Weindling [47] observed that the *T. lignorum* strain protects citrus seedlings against the *Rhizoctonia solani* pathogen through the mechanism of necrotrophic mycoparasitism. Since then, the biocontrol properties of *Trichoderma* have been extensively studied for the control of diseases caused by numerous soil phytopathogens [48]. The mechanisms by which *Trichoderma* reduces the occurrence of plant diseases include the competition for nutrients and space, synthesis of antifungal metabolites, mycoparasitism, production of lytic enzymes that degrade cell walls of fungal plant pathogens [4,49], as well as induction of plant resistance [50]. The most effective biocontrol properties are mainly attributed to the *T. virens*, *T. harzianum*, *T. koningii*, *T. pseudokoningii*, *T. longibrachiatum*, *T. asperellum*, *T. polysporum*, and *T. viride*, which have a significant impact on the development of plant diseases caused by *R. solani*, *Sclerotium rolfsii*, *Pythium aphanidermatium*, *Gaeumannomyces graminis* var. *tritici*, *Verticillium dahliae*, *Fusarium oxysporum*, and *Fusarium culmorum*, both under greenhouse and field conditions [7,24,26,51,52]. Furthermore, the application of *Trichoderma* strains to the soil increased the productivity and quality of crops of monocotyledons and dicotyledons, such as cucumbers, tomatoes, carrots, beans, corn, cotton, tobacco, millet, and ornamental grasses [53]. The stimulatory effect of *Trichoderma* on plants is probably related to their participation in the crosstalk between the growth hormones synthesized by these fungi and the defense hormones induced by them in the plant [54,55,56].

It should be noted that the *Trichoderma* fungi may also harm some areas of agriculture and human health. Their unfavorable effect in agriculture is mainly related to their ability of mycoparasitism and causes a disease entity known as green mold in mushroom (shiitake, oyster mushrooms, and champignons) cultivation [57]. The greatest losses are attributed to the *T. aggressivum* [58], *T. pleurotum*, and *T. pleuroticola* [59] genus. Several *Trichoderma* species are also included among the pathogens of cultivated plants. For instance, *T. viride* is the causative agent of onion green mold rot [41,42,60]. Recent reports indicate the occurrence of a new ear rot disease in maize in Europe caused by *T. afroharzianum* [61]. Some *Trichoderma*, especially *T. brevicompactum*, *T. atroviride*, and *T. harzianum*, can cause opportunistic infections in humans, including sinusitis, skin and liver infections, pneumonia, and stomatitis [62].

The biocontrol and biostimulation properties of *Trichoderma* directly translate into its wide application in agriculture. Due to the largest number of isolated anti-fungal bioactive compounds, *Trichoderma* is identified as the genus with the greatest biocontrol potential [18,63,64]. According to Rush et al. [64], *Trichoderma* species represent 50–60% of the fungal BCAs. Currently, at least 77 commercial *Trichoderma*-based biofungicides are available on the global market, including 7 approved by the European Commission for use in the Member States of the European Union (Supplementary Table S1) [18,64,65,66,67,68,69,70,71,72]. The above commercial preparations contain at least 36 different strains belonging to 13 species of the *Trichoderma*: *T. asperellum* (9), *T. afroharzianum* (formerly *T. harzianum*, 1), *T. atroviride* (8), *T. atrobrunneum* (1), *T. asperelloides* (1), *T. fertile* (1), *T. gamsii* (1), *T. harzianum* (7), *T. hamatum* (1), *T. polysporum* (1), *T. stromaticum* (1), *T. virens* (1), and *T. viride* (3), applied separately or in a consortium (Supplementary Table S1). It should be taken into account that due to the recent taxonomic changes within the *Trichoderma*, it is often difficult to precisely assign the strains constituting the active ingredient of biopreparations to the species [35,73,74].

## 3. Biocontrol Properties of *Trichoderma* against Fungal Phytopathogens

**Figure 2:**
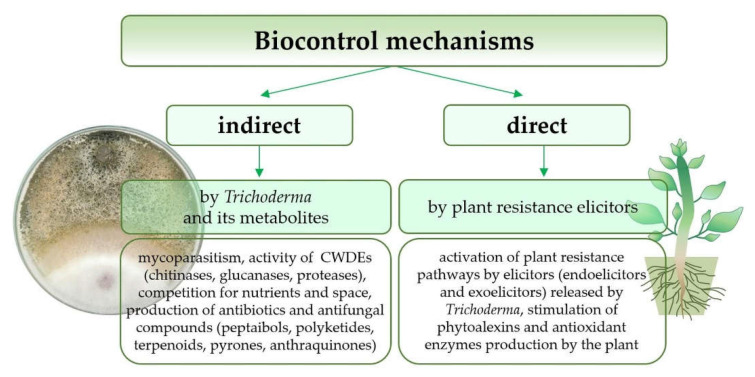
Biocontrol mechanisms used by the *Trichoderma* genus against fungal pathogens.

*Trichoderma* fungi use various complex direct or indirect mechanisms against fungal pathogens, which usually interact altogether in the biocontrol phenomenon (Figure 2) [4]. The direct impact on pathogens includes the production of cell wall degrading enzymes (CWDEs), synthesis of antibiotics, competition for space and nutrients (mainly carbon, nitrogen, and iron), and establishment of a direct parasitic relationship with the fungal pathogen [7,26,51,75]. On the other hand, *Trichoderma* indirectly induces local or systemic plant resistance through products (elicitors) released from the cell walls of the plant host (endoelicytors) and the infecting microorganism (exoelicytors) [50]. The type of mechanisms involved is often a strain characteristic and depends on the interaction type between the antagonist microorganism, pathogen, and the host plant [4,7].

### 3.1. Mycoparasitism as a Decisive Factor in Effective Biocontrol

**Figure 3:**
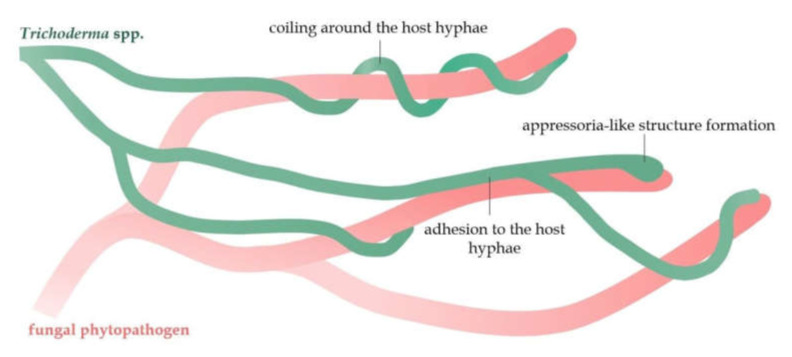
A schematic mycoparasitic interaction of the *Trichoderma* hyphae with the hyphae of fungal pathogens.

Mycoparasitism is a phenomenon in which an antagonistic fungus (mycoparasite) can parasitize on another fungus (host) [4]. The fungi of the *Trichoderma* genus are mostly classified as necrotrophic mycoparasites [76]. Over 75 *Trichoderma* species with high mycoparasitic potential have already been described [75]. The mycoparasitic effect of *Trichoderma* necrotrophs on fungal pathogens includes prey sensing and chemotaxis, adhesion to the host, and physical attack through intense branching and coiling around the host’s hyphae. Moreover, *Trichoderma* can form appressoria-like penetration structures, homologs of pathogen appressoria (Figure 3) [77,78]. Chemical attack and degradation of the pathogen’s cell wall by hydrolytic enzymes and antifungal compounds produced by *Trichoderma* is the last stage of the mycoparasitic interaction, ultimately leading to the host death [76,77].

Numerous *Trichoderma* genes encoding proteases (especially belonging to the subtilisin-like serine protease group) and oligopeptide transporters are expressed before and during the contact of various *Trichoderma* species with the host [79,80]. Moreover, it has been suggested that class IV G-protein coupled receptors (GPCRs), sensors for oligopeptides and compounds released from phytopathogen cell walls by the action of protease enzymes, are involved in the pathogenic host sensing [75]. Further signal transduction occurs through the conserved signaling cascade of G proteins, which contains three subunits: Gα, Gβ, and Gγ. *T. atroviride* mutants with a Gα subunit dysfunction completely lost their mycoparasitic capacity, decreased chitinolytic activity, and inhibited the production of the antifungal compound, 6-pentyl-α-pyrone [75,81,82]. In addition, there is biochemical evidence for the participation of Gα in the coiling of *Trichoderma* hyphae around the host hyphae. The presence of G-protein activators (mastoparan and fluoroaluminate) was found to increase the coiling of antagonist hyphae around nylon fibers [7]. Other than that, mitogen-activated protein kinases (MAPKs), especially pathogenicity MAPK (TmkA and Tmk1), are involved in signal transduction pathways in *Trichoderma* during mycoparasitism [75,83]. The removal of TmkA reduced the antagonistic interactions of *T. virens* towards *S. rolfsii*, *R. solani*, and *Pythium ultimatum* [75]. Moreover, the pathogenic fungi lectins and proteins containing cellulose-binding modules from *Trichoderma* hyphae may cooperate in the adhesion phase of mycoparasites to the victim’s hyphae [75].

The necrotrophic mycoparasitic effect was documented in the study by Błaszczyk et al. [49] during the interaction of *T. atroviride* AN240 and *T. viride* AN255 strains with *F. avenaceum* and *F. graminearum*, respectively. Similarly, the presence of the mycoparasites, *T. harzianum* [84] and *T. virens* [85], was found to be effective towards the hyphae of the *R. solani*. Moreover, effective mycoparasitic activity (coiling around the host hyphae and cell wall degradation) and changes in hyphal morphology were observed during the interaction of *T. cerinum* with the *F. oxysporum* [85]. Furthermore, the in vivo studies showed wilt disease suppression of chickpeas by the mycoparasitic *T. cerinum* Gur1 strain [86]. In their studies, Sánchez-Montesinos et al. [58] found the effective coiling of the *T. aggressivum* f. *europaeum* hyphae around the *Botrytis cinerea*, *Sclerotinia sclerotiorum*, *Fusarium solani* f. *cucurbits*, *Pythium aphanidermatum*, *R. solani*, and *Mycosphaerella melonis*.

Mycoparasitism by *Trichoderma* is not only limited to pathogenic hyphae of the host, and these fungi may use an additional biocontrol mechanism by acting on the pathogen’s conidia. The hyphae of the *Trichoderma* TkZ3A0 strain (with high similarity to the *T. koningiopsis* species) were able to parasitize on phialides with macroconidia of the *F. culmorum* phytopathogen (isolated from winter wheat with severe fusariosis symptoms) and showed clear chemotaxis and adhesion to their structures [26]. Changes in the morphology of macroconidia and inhibition of their germination (mycostasis) were observed, even in the early stages of the interaction [26].

### 3.2. The Production of Cell Wall Degrading Enzymes (CWDEs)

The production of CWDEs is an integral part of the final stage of mycoparasitism [4]. The fungal cell wall is mostly composed of 90% polysaccharides: chitin (a polymer of N-acetylglucosamine), β-(1,3)-, β-(1,4)- and β-(1,6)-glucans (composed of D-glucose units linked via β-glycosidic bonds), α-glucans, chitosan, mannan, and galactomannan, as well as proteins [87,88,89,90,91]. *Trichoderma* strains are mainly characterized by the ability to secrete a set of extracellular enzymes, including chitinases, β-(1,3)-, β-(1,6)-glucanases, and proteases, which hydrolyze the main components of the pathogen’s cell wall [4,15].

Chitinases are the most important group of lytic enzymes synthesized by *Trichoderma* fungi, hydrolyzing the β-glycosidic links between the C1 and C4 carbon of two adjacent N-acetylglucosamines in the chitin chain [92]. Most fungal chitinases belong to the glycoside hydrolase family 18 (GH18) with a molecular weight of 20 to 90 kDa [93,94]. The chitinolytic activity of *Trichoderma* results from the presence of genes (such as *ech42*, *ech30*, *chit33*, *chit36*, *chit42*, *nag1*, and *chit18-13*) encoding chitinases synthesis, the great variety of which is responsible for the effective biocontrol against a wide range of fungal phytopathogens [4,7,95]. Interestingly, the deletion of some chitinase genes of *Trichoderma* did not result in the loss of mycoparasitic and biocontrol capacity, probably due to the large reservoir of compensatory genes [75].

Glucanase enzymes degrade the cell walls of pathogenic fungi through two sets of enzymes, exo-β-glucanases, and endo-β-glucanases [91,96]. Presumably, the genes encoding this type of enzyme are over-represented in the genomes of *Trichoderma* compared to the genomes of other related fungi [75]. The description of the *T. harzianum* and *Fusarium solani* transcriptome interaction showed an overexpression of the *bgn13.4* gene encoding β-(1,3)-glucanase during the later stages of the mycoparasitism [97]. Additionally, the deletion of the *tvbgn3* gene encoding the enzyme β-(1,6)-glucanase, decreased the biocontrol potential of *T. virens* towards *Pythium ultimum* [98]. Increased chitinolytic and glucanolytic activity was demonstrated by the *Trichoderma* TkZ3A0 isolate in liquid cultures containing the lyophilized cell wall of the pathogenic *F. culmorum* strain, as the only carbon source [26]. Similarly, the *Trichoderma* ThJt1 isolate showed maximum enzymatic activity in the presence of chitin and *S. rolfsii* cell wall [99]. The addition of colloidal chitin and yeast extract as a carbon and nitrogen source, respectively, significantly increased the chitinolytic activity of *T. asperellum* strains [100]. This indicates an enhanced production of hydrolytic enzymes in the presence of a fungal pathogen.

In addition to chitinases and glucanases, a decisive role in the mycoparasitism process is played by proteolytic enzymes, catalyzing the hydrolysis of peptide bonds in proteins [77,101]. Prb1 protease synthesized by the *T. harzianum* was shown to play a crucial role in biological control. The *prb1* transformants were shown to be up to five times more effective against *R. solani* [77,102]. In a further study, the overexpression of the *T. virens* extracellular serine protease gene *tvsp1* increased the protection of cotton seedlings against *R. solani* by 15–32%, compared to the wild-type strain [103]. The aspartic protease P6281 secreted by the *T. harzianum* significantly inhibited spore germination and the growth of plant pathogenic fungi (especially *B. cinerea* and *R. solani*), as well as reduced the occurrence of cucumber, apple, and orange gray mold [104]. Furthermore, based on microscopic analyses, the effective degradation of the *B. cinerea* cell wall was found as a result of the P6281 protease action [104]. It is assumed that proteases produced by the *Trichoderma* species regulate the expression or action of other hydrolytic enzymes involved in mycoparasitism. For instance, transformants from *T. harzianum* that simultaneously overexpressed the β-1,6-glucanase gene *bgn16.2* and *papA* (a gene that encodes an extracellular aspartyl protease) showed a 30% increase in β-1,6-glucanase activity compared to *bgn16.2* single transformants [104,105].

### 3.3. The Production of Antibiotics and Other Antifungal Compounds

The *Trichoderma* species have been found to produce numerous secondary metabolites, over 370 of which belong to different classes of chemical compounds with strong antagonistic properties [4,29,106]. Most *Trichoderma* strains produce non-volatile and volatile organic compounds (VOCs), the most important of which are peptaibols and polyketides [91].

*Trichoderma* is considered to be one of the richest sources of peptaibols, and over 80% of entries in the “Peptaibiotics Database” belong to different species of this genus [107]. Peptaibols are defined as polypeptide antibiotics with a molecular weight of 500 to 2200 Da, rich in non-proteinogenic amino acids, especially alpha-aminoisobutyric acid (Aib) and isovaline (Iva). These compounds are also characterized by the presence of an acylated N-terminus and amino alcohols (e.g., phenylalaninol or leucinol) at the C-terminal residue [34,108,109]. Peptaibols are produced by non-ribosomal peptide synthetases (NRPSs) [91], and three main gene-encoding NPRSs, *tex1*, *tex2*, and *tex3*, were identified in the *Trichoderma* genomes [75]. *Trichoderma* species synthesize various antibiotics of this group. For instance, 17 biotechnologically and agriculturally important species from the Longibrachiatum Clade, (e.g., *T. citrinoviride*, *T. longibrachiatum*, *T. pseudokoningii*, and *T. reesei*) produce several new peptaibols, mainly related to trichobrachins, suzukacillins, trichoaureocins, trichocellins, longibrachins, trichokonins, trichosporins, alamethicins, and brevicelsins [33]. Moreover, *T. harzianum* has been found to synthesize trichorzins (HA, MA, and PA), harzianins, trichotoxin, and trichokindins [110]. Other than that, *T. atroviride* releases peptaibols, such as atroviridins A–C and neoatroviridins A–D, while *T. viride* produces trichotoxins A and B, trichodecenins, trichorovins, and trichocellins [91,110]. A study conducted by Tamandegani et al. [34] found that *T. asperellum* and *T. longibrachiatum* strains increased peptaibol production (trichotoxins and longibrachins) during in vitro interactions with fungal plant pathogens *F. moniliforme*, *F. culmorum*, *F. graminearum*, *F. oxysporum*, *Alternaria solani*, and *R. solani*. This study also indicated a significant increase in the peptaibol synthetase *tex1* expression during the interaction of *T. asperellum* with *R. solani*. Moreover, three analogs of peptaibol trichogin completely inhibited the growth of phytopathogens, *B. cinerea*, *F. graminearum*, and *Penicillium expansum*. After foliar application, these analogs effectively (even by 95%) reduced the symptoms of a bean, grapevine, and ripe grape disease caused by *B. cinerea* [111].

Additionally, the *Trichoderma* species show the ability to produce polyketides, a structurally diverse group of biologically active compounds derived from bacteria, fungi, and plants [112]. The polyketides include antibiotics (e.g., tetracyclines and macrolides), mycotoxins, and pigments [105]. Polyketides are synthesized from simple units such as acetyl-CoA and malonyl-CoA by polyketide synthases (PKSs) [113]. Although *Trichoderma* genomes are abundant in PKS-encoding genes, only a few researchers have focused on the genetics and polyketide biosynthesis by these fungi. Detailed phylogenomic analysis of the PKS-encoding genes of *T. reesei*, *T. virens*, and *T. atroviride* showed that most of the polyketide synthases belong to the lovastatin/citrinin or fumonisin clades, and are present as orthologs in all three species [114]. Further, the PKS gene *pks4* from *T. reesei* is responsible for green pigmentation and the stability of the conidial cell wall, as well as the antagonistic capacity against other fungi [115]. Moreover, two PKS-encoding genes, *pksT-1* and *pksT-2*, from *T. harzianum* were differentially expressed during contact with *R. solani* and *F. oxysporum* phytopathogens [116].

Numerous *Trichoderma* species produce secondary metabolites belonging to the group of terpenoids, pyrones, anthraquinones, and epipolythiodioxopiperazines (ETPs) [109,117]. The terpenoids identified in *Trichoderma* include tetracyclic diterpenes (e.g., harziandion), sesquiterpenes, such as trichothecenes (trichodermin and harzianum A), as well as triterpene viridin [109]. Furthermore, *T. viride*, *T. harzianum*, and *T. koningii* species produce the volatile antibiotic 6-phenyl-α-pyrone (6PAP), which is responsible for the characteristic coconut smell and the biological control against *F. oxysporum* [29]. Importantly, *T. harzianum*, *T. viride*, and *T. aureoviride* produce numerous anthraquinone pigments, such as pachybasin, chrysophanol, and emodin, which have strong antagonistic properties against pathogenic fungi [118], and mediate in mycoparasitic coiling [119]. The best-known ETP is gliotoxin, named after the fungus *Gliocladium fimbriatum* (later identified as *T. virens*) from which gliotoxin was originally isolated [109]. Gliotoxin exhibits immunosuppressive activity and is a virulence factor of the human pathogen *Aspergillus fumigatus*, but also plays a crucial role in biological control due to its strong fungitoxic activity [120]. *T. virens* mutants with a deletion of the *gliP* gene encoding gliotoxin synthesis showed an impaired mycoparasitic capacity against *P. ultimum* and *S. sclerotiorum* [121]. Moreover, *Trichoderma* fungi, especially *T. harzianum*, produce harzianic acid (belonging to the subgroup of tetramic acids) with greatly interesting properties due to its antifungal activity, stimulation of plant growth, and chelating abilities [122].

### 3.4. Competition for Nutrients and Space

The antagonistic fungi can deprive pathogens of space and nutrients by colonizing a common habitat, i.e., plant tissues, rhizospheres, or phyllospheres [4]. This depends on their properties, the degree of colonization of the host plant, and adaptation to the environmental conditions in which they live [4,91]. As for the successful competition with pathogens for niches and nutrients, *Trichoderma* should have effective plant colonization strategies and be abundant in a niche where competition with other fungi occurs [4]. Species belonging to this genus are widely known for their rapid growth and are considered to be aggressive competitors. They quickly colonize various substrates and eliminate slower-growing pathogens [123]. *Trichoderma* fungi are characterized by a particularly high growth rate on glucose and sucrose [26]. At the same time, it is worth emphasizing that there is a wide range of substrates that these fungi can utilize as the only source of C and energy, which allows them to effectively use a variety of rhizodeposites, both simple sugars and polymeric carbohydrates that are components of cell walls [26,91]. *Trichoderma* species have a much better ability to mobilize and uptake nutrients from the soil compared to other microorganisms [24]. This process is related to the biosynthesis of gluconic, citric, and fumaric organic acids, which lower the soil pH and increase the solubilization of phosphates and microelements (iron, manganese, or magnesium) [124].

**Figure 4:**
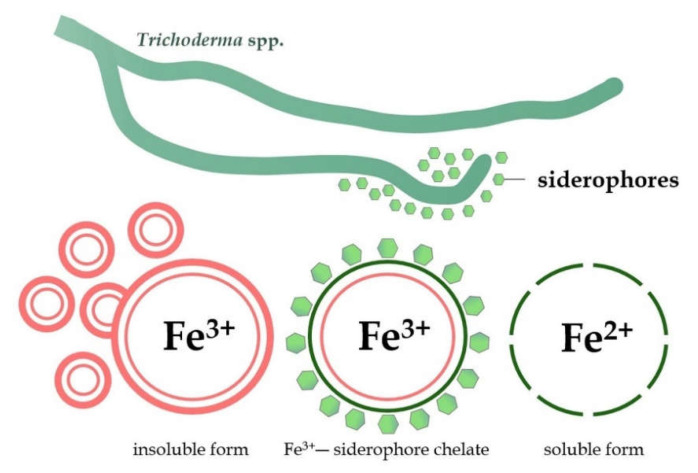
Transformation of the insoluble form of iron (Fe^3+^) into a soluble and easily assimilate form (Fe^2+^) by siderophores produced by *Trichoderma* fungi.

The greatest importance in a relation to the competition phenomenon in *Trichoderma* is attributed to siderophores, a low molecular weight (less than 10 KDa) chelator compound with a high affinity for iron (Fe), produced under iron-deficiency stress [125,126]. The Fe ions are cofactors for many enzymes and are an important factor for the proper growth and development of plants, but also many microorganisms [127]. The microbial siderophores are usually divided into three groups, hydroxamate, catecholate, and carboxylate, based on the chemical nature and coordination sites with iron [128]. Generally, fungi synthesize hydroxamate-type siderophores, such as coprogens, ferrichromes, and fusarinines, which share a common structural unit, N5-acyl-N5-hydroxyornithine [109,129]. Under neutral pH conditions and in the presence of oxygen, iron is present in the soil mainly in the form of Fe^3+^. In the aerobic environment, Fe tends to form insoluble iron oxides, which makes it unavailable for plants [127]. Siderophores can bind with insoluble iron (Fe^3+^) and then transform it into a soluble form of Fe^2+^, easily assimilated by plants and microorganisms (Figure 4) [130].

*Trichoderma* can inhibit the growth and activity of target soil pathogens by depriving them of iron sources from a common niche [131]. In vitro studies of the interaction between *T. harzianum* and *Fusarium acuminatum*, *Alternaria alternata*, and *Alternaria infectoria* pathogens showed that nutrient starvation caused the death of the studied fungi [132]. Moreover, the iron competition was described as one of the key factors in the antagonism of *T. asperellum* against *F. oxysporum* f. sp. *lycopersici* and the protection of tomato plants against wilt disease [133]. It was also reported that *Trichoderma* can effectively compete for simple and complex carbon substrates with phytopathogens of the genera *Colletotrichum* sp., *Botrytis* sp., *Verticillium* sp., and *Phytophthora* sp. [123]. Further, the enzymes that mediated competition for nutrients and space were responsible for the potent inhibitory effect of *Trichoderma* on *B. cinerea*, *F. graminearum*, and *Macrophomina phaseolina* pathogens [134].

### 3.5. The Induction of Plant Resistance in Response to Biotic Stress

The ability of *Trichoderma* strains to colonize plant roots and establish a robust and stable relationship with them is particularly crucial in biological plant protection. Fungi belonging to this genus can indirectly affect pathogenic microorganisms via plants by inducing their local or systemic defense mechanisms [91,109]. The induction of plant resistance is a consequence of the action of various elicitors (inducers of the defense response) released from the cells of the microorganisms (exoelicitors) and plant tissues (endoelicitors). The elicitors are classified into two groups: (1) race specific elicitors that trigger gene-to-gene type defense only in specific host cultivars, and (2) released from pathogenic and non-pathogenic strains general elicitors that trigger non-race specific defense both in host and non-host plant [135,136]. Different classes of elicitors were characterized, including oligosaccharides (e.g., glucans and chitins, and oligogalacturonides), proteins and peptides (e.g., endoxylanase and elicitins), glycopeptides and glycoproteins (e.g., glycopeptide fragments of invertase), glycolipids (e.g., lipopolysaccharides), and lipophilic compounds (e.g., fatty acids) [135,136]. The activation of signal transduction pathways by elicitors lead to the physical, biochemical, and molecular changes in plants, such as ion flow across the membrane, the production of reactive oxygen species (ROS), creating a physical barrier to prevent the spread of phytopathogens (callose deposition, reinforcement of plant cell wall), and the synthesis of different defense compounds (for instance, phytoalexins, volatile organic compounds, enzymes, and phytohormones) [136,137,138,139].

**Figure 5:**
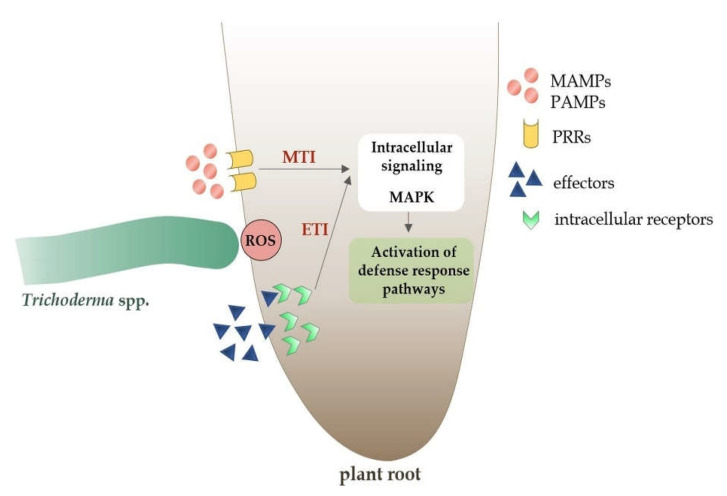
Plant defense response pathways to microbe-associated molecular pattern (MAMPs) molecules and effectors from non-pathogenic *Trichoderma* spp.

Various plant species, both monocotyledonous and dicotyledonous, show an increased activity of the immune response in the presence of non-pathogenic fungi of the *Trichoderma* [131]. The plant defense response is primarily based on the recognition of conserved domains, such as the microbe-associated molecular pattern (MAMP) or pathogen-associated molecular pattern (PAMP) [140]. These domains induce two types of innate immunity in plants: (1) MAMP-triggered immunity (MTI)/PAMP-triggered immunity (PTI) and (2) effector-triggered immunity (ETI) (Figure 5) [26].

Both transmembrane pattern recognition receptors (PRRs) and intracellular receptors recognize particular molecular patterns of MAMP/PAMP and ETI molecules, initiating local MTI/PTI or ETI immunity [91,141]. In the case of the ETI-type immune response, it is usually stronger than MTI/PTI and leads to programmed cell death as a result of hypersensitive response (HR) activation [142]. Mitogen-activated protein kinases (MAPKs) transmit information from receptors to plant cells and initiate the systemic cascade of the immune response (Figure 5) [143].

**Figure 6:**
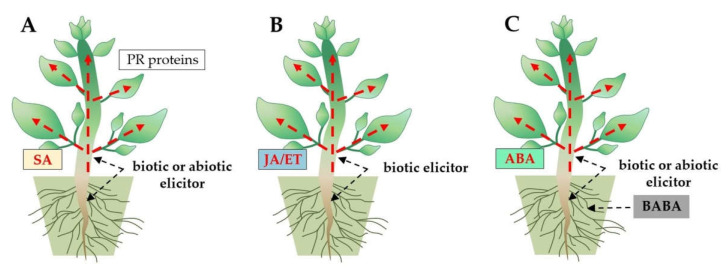
Three types of induced resistance in plants: (**A**) systemic acquired resistance (SAR), (**B**) induced systemic resistance (ISR), and (**C**) induced resistance (IR), trigged by *Trichoderma* species (biotic elicitor). Signaling molecules, such as phytohormones salicylic acid (SA), jasmonic acid (JA)/ethylene (ET), and abscisic acid (ABA), are involved in SAR, ISR, and IR immunity, respectively.

Three types of induced resistance in plants are activated as a result of the MTI and ETI immune pathways induction by *Trichoderma*: (1) systemic acquired resistance (SAR) effective against biotrophic pathogens, (2) induced systemic resistance (ISR) effective against necrotrophic pathogens, and (3) induced resistance (IR) effective in defense against biotrophic and necrotrophic pathogens and some abiotic stress factors [144,145,146,147]. The SAR is characterized by the expression of pathogenesis-related (PR) protein genes and the production of salicylic acid (SA) as a signaling molecule [145,147]. In turn, jasmonic acid (JA) and ethylene (ET) are crucial signaling molecules in ISR-type immunity [146,147]. The IR immunity is activated by β-aminobutyric acid (BABA) and involves abscisic acid (ABA) as a signaling molecule [144] (Figure 6).

The increase in the plant resistance marker’s (enzymes and metabolites) activity, as a result of the action of *Trichoderma* elicitors, indicates the induction of signaling pathways of the plant’s defense response. The inoculation of wheat seeds with *Trichoderma* TkZ3A0 strain conidia significantly increased the activity of the phenylalanine (PAL) and tyrosine lyase (TAL), catalase (CAT), guaiacol peroxidase (GPX), as well as glucanase and chitinase (PR proteins) in the plant stems and roots [26]. The PAL, a principal enzyme of the phenylpropanoid pathway, plays a crucial role in phytoalexins production and lignin biosynthesis [26,148]. On the other hand, peroxidase, catalase, and superoxide dismutase are produced by plant cells to protect against damage and oxidative stress [136]. The characteristics of the induced resistance against fungal pathogen *Colletotrichum truncatum* were demonstrated by chili pepper (*Capsicum annum* L.) treated with *T. harzianum* (as a foliar spray) and *T. asperellum* (as seed treatment) strains [149]. Moreover, the application of *T. harzianum* reduced disease development and enhanced the resistance of the grapevine against *Plasmopara viticola* [150]. In another report, *T. longibrachiatum* mediated plant systemic resistance to *B. cinerea* challenge through the activation of signaling pathways associated with the phytohormones JA/ET and SA in cucumber [151].

Priming adaptive strategy is particularly promising for the effectiveness of the resistance induction method against pathogens. The characteristic feature of this strategy is that a plant pre-inoculated with an elicitor can activate one or several defense pathways more rapidly and robustly after infection by pathogens than non-induced plants [152,153]. The plant colonization by *Trichoderma* species can prime defense response, enabling robust plant reactions to further pathogen attack [154].

## 4. The Effect of *Trichoderma* in Enhancing Plant Tolerance to Abiotic Stress

The presence of *Trichoderma* in the rhizosphere and plant tissues leads to increased plant tolerance to both biotic and abiotic stresses [155]. The most harmful environmental stresses that menace crop yield are drought, salinity, heavy metal accumulation, and extreme temperatures [156,157,158]. Adverse environmental conditions can disrupt the photosynthesis process, generate high ROS content, affect the translocation of nutrients, and modify plant hormonal balance, causing cell damage and plant necrosis [141]. However, the inoculation of tomato seedlings with *T. harzianum* under salinity and drought stress conditions resulted in the maintenance of photosynthetic efficiency and effectively reduced ROS accumulation [159]. Moreover, inoculation of wheat seedlings with *T. longibrachiatum* strain conidia enhanced the tolerance of wheat to salt stress and significantly increased the concentration of antioxidant enzymes [160]. It was found that the rapeseed (*Brassica napus* L.) plants inoculated with *T. parareesei* had a high salt and oxidative stress tolerance. Upon the root inoculation of this strain, rapeseed plants showed a significantly greater yield compared to non-inoculated plants. In addition, *T. parareesei* enhanced the tolerance to salinity and drought stress in rapeseed by increasing the expression of *ACCO1*, *NCED3*, and *PYL4* genes related to the ABA and ET hormonal pathways [157]. Durum wheat inoculated with *T. harzianum* showed a higher tolerance to moderate drought stress by 52% under optimal nitrogen fertilization; however, nutrient availability in the soil and environmental conditions significantly influenced this response [161].

Furthermore, *Trichoderma* was found to increase the plant’s tolerance to low-temperature stress. The inoculation of tomato (*Solanum lycopersicum* L.) plants with *T. harzianum* strain efficiently alleviated the adverse effects of cold stress leading to an increased fresh and dry weight of shoots and roots, as well as improved photosynthesis, growth rate, and leaf water content. In addition, *TAS14* and *P5CS* gene-encoding enzymes and metabolic proteins that mediate plant tolerance to low-temperature stress were up-regulated with *T. harzianum* inoculation [162]. On the other hand, *T. koningii* proved to be beneficial in imparting heat stress tolerance to the tomato plants through the enhanced modulation of antioxidants. The application of this strain reduced the ROS accumulation and protected the plant cells from oxidative damage under high-temperature stress [163].

## 5. *Trichoderma* as a Biocontrol Agent against Other Plant Pathogenic Organisms

Numerous strains of *Trichoderma* have been developed as biocontrol agents against plant diseases caused by bacteria, nematodes, and insects [158,164]. There are more than 200 species of plant pathogenic bacteria, among which *Agrobacterium*, *Pseudomonas*, *Erwinia*, *Ralstonia*, and *Xanthomonas* cause the most severe crop losses worldwide [165]. The antibacterial activity of *Trichoderma* is most often attributed to the secretion of secondary metabolites, the most important of which are peptaibols, gliotoxin, polyketides, gliovirin, and pyrones [165,166]. *T. pseudoharzianum* and *T. asperelloides* showed a strong inhibitory effect on the growth of the serious bacterial pathogen of tomato plants, *Ralstonia solanacearum*. Changes in the morphology of *R. solanacearum* cells (the rupturing of cell walls, the disintegration of the cell membrane, and leakage of cell contents) were observed as a result of the action of various secondary compounds from *Trichoderma* [165]. Moreover, *T. hamatum* synthesized the bioactive volatile secondary metabolite, 6PAP, which effectively inhibited the growth of *Acidovorax avenae*, *Erutimacarafavora*, and *Xanthomonas campestris* [167]. Sarsaiya et al. [168] demonstrated for the first time that the endophytic strain of *T. longibrachiatum*, isolated from the medicine plant *Dendrobium nobile*, produced the dendrobine compound responsible for enhancing human immunity, preventing metastatic cancer, and therapeutic effects on Alzheimer’s disease. Other than that, dendrobine exhibited strong toxic activity against bacterial plant pathogens, such as *Bacillus subtilis*, *Bacillus mycoides*, and *Staphylococcus* sp. [168]. This indicates the potential applications of *Trichoderma* metabolites in both agricultural and medical fields.

Plant-parasitic nematodes (PPNs) represent a vital threat to agricultural production. Worldwide crop damage caused by plant nematodes has been estimated at around 12% [169]. *Trichoderma* was found to have strong nematicidal activity against PPNs. The biocontrol action of *Trichoderma* against phytoparasitic nematodes occurs through the direct parasitism of eggs and larvae as well as the production of hydrolytic enzymes (chitinases and proteases) and secondary metabolites (volatile and non-volatile) that destroy the cuticle of nematodes. Furthermore, *Trichoderma* hyphae may form a physical and chemical protective barrier over the plant roots [170]. The application of *T. harzianum* to tomato seeds greatly reduced the severity of disease caused by the root-knot nematode *Meloidogyne javanica.* In this study, *T. harzianum* efficiently penetrated the nematode mass matrix and decreased egg hatching. In tomato plants, defense enzymes activity (peroxidase, polyphenol oxidase, and PAL) was significantly increased [171]. Moreover, *T. asperellum* and *T. harzianum* have been reported to inhibit the penetration of *Pratylenchus brachyurus* in soybeans by producing non-volatile compounds with nematicidal activity [170].

Recent studies demonstrated that *Trichoderma* can enhance plant protection against insect pests, such as aphids, thrips, moths, and caterpillars [172]. *Trichoderma* directly affects pests through some antagonistic mechanisms, the most important of which are parasitism and the production of insecticidal secondary metabolites, antifeedant compounds, and repellents. Moreover, *Trichoderma* has been reported to directly reduce the detrimental effect of these pathogens by the activation of plant defense mechanisms [173]. *T. longibrachiatum* showed an entomopathogenic effect on the development of *Leucinodes orbonalis*, one of the major pests of *Solanum melongena* (eggplant) [174]. Further examination indicated that there was an intense parasitic growth of the *T. gamsii* hyphae across the lower abdomen and respiratory ostiole of the *Delia radicum* larva [175]. Other than that, *T. harzianum*, *T. asperellum*, and *T. hamatum* showed pathogenic potential against *Cetarovacuna lanigera*, a destructive insect pest of sugarcane that is responsible for reducing the quality, yield, and sugar content [176].

## 6. Plant Growth-Promoting Properties of *Trichoderma*

*Trichoderma* is often associated with the root ecosystems of the host plants. Therefore, *Trichoderma* is usually defined as the genus of symbiotic, opportunistic, and avirulent microorganisms that colonize the roots and stimulate plant growth through mechanisms similar to those used by mycorrhizal fungi [7]. The beneficial effects of *Trichoderma* species on plants include the promotion of their growth, improvement to root structure and condition, enhancement of seed germination and viability, as well as increased photosynthesis efficiency, flowering, and yield quality [177]. The most important stimulating factor at almost all stages of plant growth and development is the synthesis of phytohormones and phytoregulators [26,178].

### 6.1. Plant Root Colonization

Numerous rhizosphere *Trichoderma* species can colonize the root surfaces of both monocotyledonous and dicotyledonous plants, leading to significant changes in plant metabolism [179]. Colonization by the *Trichoderma* species involves the recognition of host plant, attachment and penetration of plant roots, and resistance of *Trichoderma* to metabolites produced by the plants in response to invasion by foreign organisms (pathogenic or non-pathogenic) [7]. Due to the colonization of a wide range of host plants, *Trichoderma* presumably developed effective strategies to overcome plant defense mechanisms [179].

**Figure 7:**
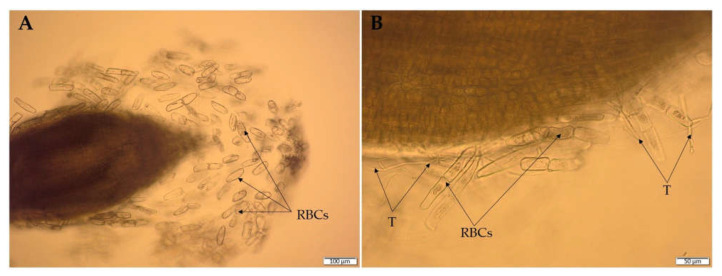
Wheat root border cells (RBCs) (**A**) and RBC colonization (**B**) by *Trichoderma* DEMTkZ3A0 hyphae (T) observed in microcultures on glass slides using an Olympus BX53 Upright Microscope equipped with a Olympus XC30 camera.

Numerous studies confirmed the ability of *Trichoderma* not only to colonize the rhizosphere soil, but also to colonize the root surface [180,181]. The root colonization by *Trichoderma* was previously observed mainly in the root hair and elongation zone. However, recent studies confirmed the colonization of the root cap border cells (RBCs) zone by these fungi [26]. The RBCs play a crucial role in the interaction between plants and soil microorganisms [182,183]. The RBCs are released into the soil environment from the outer layer of the cap cells during root elongation (Figure 7A). It is assumed that these cells and the associated root exudates constitute a valuable source of nutrients and biologically active compounds for microorganisms [182]. Moreover, RBCs influence the composition and structure of the microbial rhizosphere community and act as attractants for microorganisms, facilitating the interaction with the plants and root colonization [182,184]. Simultaneously, RBCs are considered a crucial agent in plant protection against various biotic and abiotic stresses [185]. The ability of *Trichoderma* to colonize RBCs, as demonstrated for the wheat roots inoculated with DEMTkZ3A0 strain conidia (Figure 7B) [26], indicates an important role of these fungi in the indirect defense mechanism against phytopathogens through the formation of a mantle-like structure.

It was found that the recognition and adhesion of *Trichoderma* to the root surface of the host plants is mediated by hydrophobins, which play a fundamental role in cell communication, fungal morphogenesis (including infectious structures), and the adhesion of hyphae to hydrophobic surfaces [186,187]. For instance, the *T. harzianum* gene, *qid74*, encodes a cysteine-rich protein, involved in the adhesion of fungal hyphae to the tomato root system [188]. On the other hand, the deletion of the *TasHyd1* hydrophobin gene from *T. asperellum* resulted in a change in the hyphae wettability and serious impairment in root attachment and colonization [187]. *Trichoderma* also produces proteins with a high affinity for cellulose, among which cerato-platanins and swollenins are crucial for the effective root colonization of plants [180,189]. *T. atroviride* strains that overexpressed the *Taswo1* swollenin gene showed improved colonization of pepper (*Capsicum annuum* L.) roots and a stronger plant growth-promoting effect [189]. Furthermore, the root colonization by *Trichoderma* is closely related to the production of cellulolytic, proteolytic, pectinolytic, and xylanolytic plant cell wall degrading enzymes (PCWDEs), enabling the colonization of the root epidermis and cortex area [91,179,190]. Moreover, PCWDEs may release fragments of the plant cell wall, acting as plant resistance endoelicitors [26].

The plant-derived sucrose, a predominant resource supplied to *Trichoderma* cells, is associated with the rapid growth of fungi and facilitated root colonization [75,191]. A similar effect is shown by carbon substrates (mono- and disaccharides) secreted by the mucigel layer of the roots during the mycorrhiza process [75]. In the presence of plant sucrose, the intracellular invertase from *T. virens* (TvInv) was shown to play an important role in the mechanisms controlling the symbiotic association with maize (*Zea mays* L.) roots [191]. Some important solute transporters, such as di/tripeptide transporter and the permease or intracellular invertase system involved in the obtaining and transport of root exudates, were described in the *Trichoderma* genomes [192].

### 6.2. The Synthesis of Phytohormones and Metabolites Influencing the Phytohormonal Balance

The microbial production of phytohormones and phytoregulators is one of the direct mechanisms contributing to the rapid and stable colonization of soil by microorganisms and their promoting effect on plant growth [193,194]. Furthermore, the colonization of plant tissues by phytohormone-synthesizing soil microorganisms affects the hormonal balance of plants and their interaction with microorganisms [195,196]. Microbial phytohormones play crucial roles in agriculture with a growing interest in their industrial production, especially with the use of fungal cultures [197]. Phytohormones participate in the regulation of complex and interrelated plant immune signaling pathways, ensuring a rapid defense response and adaptation to various environmental conditions [198]. Several representatives of the genus *Trichoderma* are characterized by the ability to produce phytohormones (auxin and gibberellin) and phytoregulators, including the 1-aminocyclopropane-1-carboxylic acid (ACC) deaminase enzyme, which regulates the ethylene biosynthetic pathway [26,198,199]. The synthesis of the indole-3-acetic acid (IAA) hormone by *Trichoderma* results in the enhancement of the colonization capacity of the rhizosphere, rhizoplane, and roots of monocotyledons and dicotyledons, which was observed during the interaction of the *T. harzianum* mutant with a cucumber [200]. Moreover, the growth stimulation of cucumber roots was enhanced by the *T. asperellum* Q1 strain, capable of producing a complex of growth hormones (IAA, gibberellin, and ABA) [201]. Furthermore, the reinforcement growth of wheat seedlings was observed upon *Trichoderma* strain inoculation, capable of producing IAA, gibberellin, and ACC deaminase [26].

#### 6.2.1. The Production of Auxin Phytohormone Indole-3-Acetic Acid (IAA)

Several species of *Trichoderma* produce auxin phytohormones, especially IAA, which is crucial for most of the processes responsible for the proper growth and development of plants. It is assumed that IAA is the main factor determining competition between fungal species inhabiting the same niche [202,203]. The production of microbial IAA usually depends on the presence of its precursor, L-tryptophan. However, some microorganisms synthesize IAA in amino acid independent pathways [199]. The in vitro production of IAA by *Trichoderma* strains has been demonstrated in the presence of tryptophan [26,204]. A similar dependence was also found in the case of endophytic bacteria strains [205] and *Mortierella* fungi [199,206] with biostimulatory potential. The production of IAA by *Trichoderma* also depends on abiotic factors, such as temperature and pH [26,207].

IAA at low concentrations stimulates root elongation, while the high concentration of IAA is responsible for the proper formation of lateral and adventitious roots [26]. Upon the inoculation of *T. virens* and *T. atroviride*, the biomass production and stimulation of lateral root growth of the wild-type *Arabidopsis thaliana* seedlings were enhanced [208]. In the presence of *T. atroviride* closely related strains, the number of *A. thaliana* secondary roots increased by 64 to 90% compared to non-inoculated plants [203]. Moreover, mutations in the *aux1*, *big*, *eir1*, and *axr1* genes involved in auxin signaling and transport were found to reduce the stimulating effect of *T. virens* on plant root growth and development [208]. The synthesis of IAA by the *T. harzianum* mutant may affect the composition of the rhizosphere and rhizoplane soil and facilitate the colonization of cucumber roots and stems [200]. Other than that, the improvement of plant growth due to the action of IAA derived from the *Trichoderma* species was also reported in many other agricultural plants, including tomato [209], sorghum [204], bean [53], wheat [210], and pepper [211].

#### 6.2.2. The Production of Gibberellin Phytohormones

Gibberellins (GAs) are one of the main phytohormones, the low concentration of which affects the proper germination of seeds, the growth of plant roots and shoots, leaf expansion, and flower development [199]. These properties are responsible for the extensive application of GAs in agriculture to improve the quality of agricultural and horticultural crops [212]. Almost 136 different GA compounds are already known, while only GA_1_, GA_3_, GA_4_, and GA_7_ from the C_19_-GA group are biologically active [213,214].

Several scientific reports indicate the ability of numerous species from the *Trichoderma* to synthesize GAs [26,215,216]. After the application of *T. koningiopsis* isolates, tomato growth was significantly improved, possibly through GA action [215]. Moreover, the *T. asperellum* Q1 strain synthesizing both IAA, GA, and ABA, increased the concentration of these hormones in leaves of cucumber seedlings [201]. The accumulation of GA_3_ produced by *T. harzianum* in a combination with IAA was found to increase the plant growth-promoting effect [216]. Furthermore, the production of GA by the *Trichoderma* strain was positively correlated with the synthesis of phytohormone IAA and phytoregulator ACC-deaminase [26].

#### 6.2.3. The Production of the ACC-Deaminase Enzyme

Plant growth stimulation also occurs in the presence of *Trichoderma* with the ability to produce the ACC-deaminase enzyme (ACCD), which lowers the ethylene (ET) levels in plants by cleaving the ET precursor ACC into α-ketobutyrate and ammonia [217]. The ET is produced by plants in a response to numerous environmental biotic and abiotic stresses [218]. This phytohormone (ET) is also involved in the regulation of various physiological processes of the plant, in part through complex interactions with other phytohormones [219]. However, high levels of ET can inhibit root elongation and growth, often leading to plant death [220,221].

Several species of *Trichoderma* fungi have the considerable ability to produce ACCD involved in promoting plant growth [26,222,223]. Rauf et al. [218] proved an increased tolerance of wheat to waterlogging stress as a result of the activity of ACCD produced by *T. asperellum*. Moreover, ACCD from *T. harzianum* positively influenced the germination and growth of maize seedlings under greenhouse conditions [223]. The genes encoding the ACCD enzyme were identified in the genomes of *Trichoderma*. The silencing of the *Tas-acdS* gene from *T. asperellum* showed a decreased ability to stimulate the root elongation of canola plants [224].

The release of ACC by plant roots into the rhizosphere soil to attract and interact with plant growth-promoting microorganisms (PGPMs) suggests an ethylene-independent function of ACC [225]. A study conducted by Viterbo et al. [224] showed that the addition of ACC to the medium as the sole nitrogen source increased the ACCD activity of *T. asperellum* T203 strain. Moreover, tomato plants pretreated with ACC proved less serious disease symptoms caused by the *V. dahliae* pathogen, compared to untreated controls. This confirms that ACC may act as a signal molecule for control defense and enhance plant resistance to diseases [225].

### 6.3. Nutrient Solubilization and Enhancement Bioavailability of Essential Elements

*Trichoderma* plays a crucial role in enhancing plant growth by the production of vitamins, increasing the solubility of nutrients contained in the rhizosphere (phosphates, Fe^3+^, Cu^2+^, Mn^4+^, ZnO), and supplementing the plant with the necessary elements (mainly nitrogen, phosphorus, potassium, and microelements) for their proper growth and yield [26,91,179,226].

Amongst all the plant nutrients, phosphorus (P) is probably present in the soil in the forms with the most limited bioavailability to plants [227]. The soil application of *Trichoderma* strains was demonstrated experimentally to increase inorganic phosphate solubilization due to extra-cellular phytase activity [228] and acidification of the soil environment by acetic, butyric, citric, and fumaric acids production [229]. The ability of *Trichoderma* to solubilize phosphates was correlated with improved beans, wheat [230], rice [231], soybean [232], and mangrove [228] growth.

A study conducted by Li et al. [233] showed an increased nutrient (P, K, Mg, and Zn) uptake by tomato plants after pre-inoculation with *T. asperellum* CHF 78 strain. Moreover, the *T. harzianum* strain produced in liquid cultures diffusible metabolites capable of reducing Fe(III) and Cu(II) [234]. On the other hand, Singh et al. [235] proved that *T. asperellum* T42 mediated the enhancement in host biomass, total nitrogen content, production of nitric oxide (NO), and the accumulation of cytosolic Ca^2+^ in tobacco.

## 7. Conclusions and Future Prospects

The *Trichoderma*─plant─pathogen interaction is a very dynamic and multi-dependent system. Detailed knowledge of the *Trichoderma* mechanisms towards plants and pathogens can significantly increase the effectiveness of their action. *Trichoderma* uses several complex direct and indirect biocontrol mechanisms, both against biotic stresses, such as wide spectrum of pathogenic microorganisms (fungi, bacteria, insects, and nematodes), and abiotic stresses—unfavorable environmental conditions.

The knowledge about the extraordinary abilities of *Trichoderma* gained in recent years contributes to the creation of biopreparations based on strains with a comprehensive and beneficial effect on plants. These *Trichoderma* preparations will find wide application in organic farming to combat plant diseases of various etiologies, where they have a chance to provide complete protection without the use of chemical pesticides. In turn, the resistance of *Trichoderma* to chemical pesticides will make it possible to combine these fungi in preparations with low concentrations of various, especially newly introduced and modified, chemical pesticides. Furthermore, *Trichoderma* has the potential to become the basis of new phytoremediation technologies due to its resistance to a variety of toxic chemicals, both organic and inorganic, and increase plant tolerance to stress factors under conditions of xenobiotic contamination. Importantly, these solutions are in line with the idea of sustainable agriculture.

In the coming years, the methods of enhancing the effectiveness and reliability of *Trichoderma* preparations will be improved through treatments increasing the competitiveness of *Trichoderma* in the rhizosphere and rhizoplane. This can be achieved through strong and stable colonization of these zones and the combination with microorganisms supporting the positive effects of *Trichoderma*, the so-called supporting strains [236,237]. For instance, the volatile compounds (VCs) from some bacteria have been shown to significantly affect the secretion of antimicrobial and antifungal compounds by *T. virens* and *T. harzianum* [238].

Fungi belonging to the genus *Trichoderma* have become an excellent model for the study of substances determining the nature of the interaction of microorganisms with the plants, i.e., effector-like molecules [239]. Research on *Trichoderma* identified several plant interaction effectors, including proteins (cerato-platanins, glycoside hydrolases, hydrophobins, and small secreted cysteine-rich proteins), secondary metabolites (lactones, peptaibols, polyketides, terpenes, trichothecenes, VOCs, and phytohormones), and small (20−30 nucleotide long) non-coding RNAs. Direct evidence for the role of beneficial microorganisms RNAs on the suppression of plant immunity to establish a symbiotic relationship is sought. *Trichoderma*, through the production of RNAs, establishes symbiotic and non-pathogenic associations with plants [240]. Since most symbiotic effectors are members of the same families present in phytopathogens, further intensive studies of the effector molecules produced by *Trichoderma* will explain not only the course of interaction with microorganisms that favorably affect plants, but also those that may lead to a negative impact on agriculture.

The great importance of *Trichoderma* in signaling and influencing the virulence of pathogens is associated with the ability to release an unusual universal signaling substance, N-acetylglucosamine (GlcNAc), as a result of chitinolytic activity [241]. A crucial step in a successful attack on fungal hosts is the production of hydrolytic enzymes that permeabilize and degrade the fungal cell wall [242].

The main challenge is to develop and use in *Trichoderma* research the latest, comprehensive, advanced, and at the same time cheap, fast, and effective methods of detecting and testing antagonists, combining various modes of action and causing a cascade of reactions [243,244,245,246]. The research will also be aimed at the accurate and quick estimation of the risk of using BCAs based on *Trichoderma*, their toxicity and ecotoxicity not only in in vitro conditions, but above all in natural growing conditions in vitro and in situ, before being introduced to the market as biocontrols, biostimulations or bioremediation preparations. Social fears of introducing new microorganisms with antifungal and antibacterial properties into the environment should be eliminated.

## Data Availability

Not applicable.

## Supplementary Materials

The following supporting information can be downloaded at: https://www.mdpi.com/article/10.3390/ijms23042329/s1.

## Abbreviations

6PAP	6-phenyl-α-pyrone
ABA	Abscisic acid
ACC	1-aminocyclopropane-1-carboxylate
ACCD	ACC-deaminase enzyme
Aib	Alpha-aminoisobutyric acid
BABA	β-aminobutyric acid
BCAs	Biological control agents
CAT	Catalase
CWDEs	Cell wall degrading enzymes
EGD	European Green Deal
ET	Ethylene
ETI	Effector-triggered immunity
ETPs	Epipolythiodioxopiperazines
EU	European Union
FAO	Food and Agriculture Organization of the United Nations
GAs	Gibberellins
GH18	Glycoside hydrolase family 18
GPCRs	G-protein coupled receptors
GPX	Guaiacol peroxidase
HR	Hypersensitive response
IAA	Indole-3-acetic acid
IPM	Integrated pest management
IR	Induced resistance
ISR	Induced systemic resistance
Iva	Isovaline
JA	Jasmonic acid
MAMP	Microbe-associated molecular pattern
MAPKs	Mitogen-activated protein kinases
MTI	MAMP-triggered immunity
NO	Nitric oxide
NRPSs	Non-ribosomal peptide synthetases
P	Phosphorus
PAL	Phenylalanine lyase
PAMP	Pathogen-associated molecular pattern
PCWDEs	Plant cell wall degrading enzymes
PDA	Potato dextrose agar
PKSs	Polyketide synthases
PPNs	Plant-parasitic nematodes
PR	Pathogenesis-related
PRRs	Pattern recognition receptors
PTI	PAMP-triggered immunity
RBCs	Root cap border cells
ROS	Reactive oxygen species
SA	Salicylic acid
SAR	Systemic acquired resistance
TAL	Tyrosine lyase
VOCs	Volatile organic compounds
