# Supplementary material for: Trichoderma: The Current Status of Its Application in Agriculture for the Biocontrol of Fungal Phytopathogens and Stimulation of Plant Growth
Source: Int J Mol Sci. 2022 Feb 19;23(4):2329. doi: 10.3390/ijms23042329 (PMC8875981; doi:10.3390/ijms23042329)
Supplement: Supplementary file 1 [file ijms-23-02329-s001.zip › ijms-1592305-supplementary.pdf]

Table S1. *Trichoderma*-based commercialized biocontrol agents (BCAs) against fungal phytopathogens and their application in specific plant crops.

Name of products	Species	Strain	Registered in	Protected crops	Target pathogens
**Anushka**	*T. viride*		India[a]	permitted for use on all crops	*Phytophthora* spp., *Rhizoctonia* spp., *Sclerotinia* spp.
**BF20.001**	*T. asperellum*	ICC 012	Brazil[b]	permitted for use on all crops	*Rhizoctonia solani*, *Colletotrichum* spp., *Sclerotinia sclerotiorum*
**Bhoomika**	*T. viride*	TNAU-TV-1	India[a]	all crops and trees	*Phytophthora* spp., *Rhizoctonia* spp., *Sclerotinia* spp.
**Binab T** (Binab T Wettable Powder Biorational Fungicide)	*T. polysporum*<br>*T. viride*	ATCC 20475<br>ATCC 20476	USA[c]	forest trees, ornamental shade trees, ornamental trees	*Poria carbonica*, *Lentinus lepideus*
**Bio Zenon**	*T. harzianum*	CCT 7589	Brazil[b]	permitted for use on all crops	*Rhizoctonia solani*
**Bio-Fit**	*T. harzianum*<br>*Trichoderma* spp.<br>*T. viride*		Chile[d]	permitted for use on all crops	*Fusarium* spp., *Phytophthora* spp., *Pythium* spp., *Rhizoctonia* spp., *Verticillium* spp.
**Biofort**	*T. viride*		India[a]	permitted for use on all crops	*Phytophthora* spp., *Rhizoctonia* spp., *Sclerotinia* spp.
**Bio-Hulk**	*T. asperellum*	BV-10	Brazil[b]	permitted for use on all crops	*Rhizoctonia solani*
**Bioten WP** (Bio-Tam 2.0, Tenet WP, Remedier WP, Tenet T&O)	*T. asperellum*<br>*T. gamsii*	ICC 012<br>ICC 080	USA[c], Canada[f], EU Members[e]	strawberry, pepper, tomato, ornamental plants, grape vine, cereal grains, cole crops, berries, cucurbits, leafy vegetables, legume vegetables, herbs, onions, citrus, greenhouse and nursery	*Pythium* spp., *Rhizoctonia* spp., *Phytophthora* spp., *Phoma* spp., *Verticillium* spp., *Fusarium* spp., *Sclerotinia* spp., *Armillaria* spp., *Rosellinia* spp., *Sclerotium rolfsii*, *Phaeomoniella chlamydospora*
**Bio-Traz**	*T. harzianum*		Chile[d]	permitted for use on all crops	*Botrytis cinerea*
**Bora HC**	*T. harzianum*	KRL-AG2	Canada[f]	greenhouse crops, greenhouse vegetables, outdoor nursery plants, agricultural field crops, strawberry, lettuce, geranium	*Pythium* spp., *Rhizoctonia* spp., *Fusarium* spp.
**Bora WP**	*T. harzianum*	KRL-AG2	Canada[f]	greenhouse crops, outdoor nursery plants, greenhouse transplants	*Pythium* spp., *Rhizoctonia* spp., *Fusarium* spp., *Botrytis cinerea*

Table S1. Continuation.

Name of products	Species	Strain	Registered in	Protected crops	Target pathogens
**BW 240 G Biological Fungicide** (Rootshield Plus Granules, Rootshield Plus Granules Biological Fungicide, Rootshield Plus+ G, Rootshield Plus+ G Biological Fungicide, Rootshield Plus+ Granules, Rootshield Plus+ Granules Biological Fungicide, Turfshield Plus G, Turfshield Plus G Biological Fungicide, Turfshield Plus Granules, Turfshield Plus Granules Biological Fungicide)	*T. virens*	G-41	USA[c]	celery, cherries, chervil, chestnut, citrus, cole crops, corn, cotton, cucumber, eggplant, endive, fennel, fruiting vegetables, garlic, grapefruit, grapes, greens, herbs, leafy vegetables, lentils, lettuce, nut trees, oak, oranges, ornamental plants, onions, parsley, peppers, plantain	*Fusarium* spp., *Phytophthora* spp., *Pythium* spp., *Rhizoctonia* spp., *Thielaviopsis* spp.
	T. afroharzianum (formerly *T. harzianum*)	T-22			
**BW 240 WP Biological Fungicide** (Rootshield Plus, Rootshield Plus WP Biological Fungicide, Rootshield Plus+ WP Biological Fungicide, Rootshield Plus Wettable Powder, Rootshield Plus WP, Rootshield Plus+ WP, Turfshield Plus WP, Turfshield Plus WP Biological Fungicide)	*T. virens*	G-41	USA[c], Canada[f]	berries and small fruits, bulb vegetables, cucurbit vegetables, fruiting vegetables, leafy vegetables, cereal grains, citrus fruits, asparagus, herbs and spices, hydroponic plants	*Pythium* spp., *Phytophthora* spp., *Rhizoctonia* spp., *Fusarium* spp. *Cylindrocladium* spp., *Sclerotinia* spp., *Thielaviopsis* spp.
	T. afroharzianum (formerly *T. harzianum*)	T-22			
**Criyagen**	*T. viride*		India[a]	permitted for use on all crops	*Botrytis cinerea*, *Fusarium* spp., *Ganoderma* spp., *Sclerotinia* spp.
**Daytona**	*T. harzianum*	ESALQ-1306	Brazil[b]	beans, strawberry, soybean	*Fusarium solani* f.sp. *phaseoli*, *Rhizoctonia solani*, *Sclerotinia sclerotiorum*
**Derma HL**	*T. harzianum*	IIHR Th-2	India[a]	permitted for use on all crops	*Alternaria* spp., *Cercospora* spp., *Fusarium* spp., *Phytophthora* spp., *Rhizoctonia* spp., *Sclerotinia* spp., *Verticillium* spp.

Table S1. Continuation.

Name of products	Species	Strain	Registered in	Protected crops	Target pathogens
**Derma WHP**	*T. harzianum*	IIHR Th-2	India[a]	permitted for use on all crops	*Alternaria* spp., *Cercospora* spp., *Fusarium* spp., *Phytophthora* spp., *Rhizoctonia* spp., *Sclerotinia* spp., *Verticillium* spp.
**Ecohume GR**	*T. viride*		India[a]	permitted for use on all crops	*Alternaria* spp., *Ascochyta* spp., *Cercospora* spp., *Fusarium* spp., *Verticillium* spp.
**Ecotrich WP**	*T. harzianum*	IBLF006	Brazil[b]	permitted for use on all crops	*Rhizoctonia solani*
**ESQUIVE WP**	*T. atroviride*	I-1237	EU Members[e]	grapevine pruning, grapevine nursery	wood decay diseases
**G-41 Technical**	*T. virens*	G-41	USA[c], Canada[f]	various agricultural, greenhouse and nursery crops, plants in residential settings (e.g., vegetables, fruit and ornamentals)	*Rhizoctonia* spp., *Fusarium* spp.
**Gaia Bio** (Biagro GEB)	*T. harzianum*	CCT 7589	Brazil[b]	permitted for use on all crops	*Rhizoctonia solani*
**Hariz**	*T. harzianum*	IIHR Th-2	India[a]	permitted for use on all crops	*Botrytis* spp., *Fusarium* spp., *Penicillium* spp., *Pythium* spp.
**Incept**	*T. hamatum*	TH382	USA[c]	compost, greenhouse soils, ornamental plants, potting soil, vegetable crops	*Botrytis* spp., *Fusarium* spp., *Phytophthora* spp., *Pythium* spp., *Rhizoctonia* spp., *Sclerotinia* spp., *Thielaviopsis* spp.
**Jaivika**	*T. viride*		India[a]	permitted for use on all crops	*Botrytis cinerea*, *Botrytis* spp., *Fusarium* spp., *Pythium* spp., *Rhizoctonia* spp., *Sclerotinia* spp., *Sclerotium* spp.
**Monitor**	*T. viride*		India[a]	permitted for use on all crops	*Alternaria* spp., *Colletotrichum* spp., *Fusarium* spp., *Phytophthora* spp., *Rhizoctonia* spp., *Sclerotium* spp., *Pythium* spp.
**Natucontrol**	*T. harzianum*		Brazil[b]	permitted for use on all crops	*Rhizoctonia solani*, *Sclerotinia sclerotiorum*, *Fusarium solani*

Table S1. Continuation.

Name of products	Species	Strain	Registered in	Protected crops	Target pathogens
**Neemoderma**	*T. viride*	TNAU-TV-1	India[a]	permitted for use on all crops	*Botrytis cinerea*, *Fusarium* spp., *Ganoderma* spp., *Pythium* spp., *Rhizoctonia* spp., *Sclerotinia* spp., *Ustilago* spp.
**Niprot**	*T. viride*		India[a]	permitted for use on all crops	fungal diseases
**Organic WP**	*T. asperellum*	URM 5911	Brazil[b]	permitted for use on all crops	*Fusarium solani* f.sp. *phaseoli*, *Rhizoctonia solani*
**Pardella**	*T. asperellum*<br>*T. harzianum*	URM 8120<br>URM 8119	Brazil[b]	permitted for use on all crops	*Rhizoctonia solani*, *Sclerotinia sclerotiorum*, *Colletotrichum* spp.
**Peak Trico**	*T. viride*		India[a]	permitted for use on all crops	*Armillaria* spp., *Fusarium* spp., *Ganoderma* spp., *Pythium* spp.
**Peak Trico-H**	*T. viride*	IIHR Th-2	India[a]	permitted for use on all crops	*Armillaria* spp., *Fusarium* spp., *Ganoderma* spp., *Pythium* spp.
**Predatox**	*T. harzianum*	IBLF006	Brazil[b]	lettuce, soybean, beans	*Rhizoctonia solani*, *Sclerotinia sclerotiorum*
**Quality**	*T. asperellum*	URM 5911	Brazil[b]	permitted for use on all crops	*Fusarium* spp., *Rhizoctonia* spp.
**Rizoderma**	*T. harzianum*	IBLF006	Brazil[b]	permitted for use on all crops	*Rhizoctonia solani*, *Sclerotinia sclerotiorum*
**ROM-Tricho**	*T. viride*		India[a]	pepper, cardamon, ginger, turmeric, banana, cabbage, potato, pomegranate and other crops	*Fusarium* spp., *Phytophthora* spp., *Pythium* spp., *Sclerotium* spp.
**ROM-Trypae mixture**	*T. viride*		India[a]	banana, cardamon, pepper, turmeric, ginger, pomegranate, all types of vegetable and other crops	*Fusarium* spp., *Pythium* spp., *Phytophthora* spp.
**Sentinel**	*T. atroviride*	LU132	New Zealand[g], Australia[h]	grapevines, tomatoes	*Botrytis cinerea*
**Sharad-Tricho**	*T. viride*	IIHR-TV-5	India[a]	wide range of crops	*Phytophthora* spp., *Pythium* spp., *Rhizoctonia* spp.
**Shocker**	*T. harzianum*	CPQBA 040-11	Brazil[b]	beans, soybean	*Rhizoctonia solani*, *Sclerotinia sclerotiorum*

Table S1. Continuation.

Name of products	Species	Strain	Registered in	Protected crops	Target pathogens
**Stimucontrol**	*T. harzianum*	CCT 7589	Brazil[b]	permitted for use on all crops	*Rhizoctonia solani*, *Sclerotinia sclerotiorum*
**Stimucontrol Evolution**	*T. harzianum*	CCT 7589	Brazil[b]	permitted for use on all crops	*Rhizoctonia solani*, *Sclerotinia sclerotiorum*
**Sustain**	*T. asperellum*		Uganda[i]	permitted for use on all crops	*Fusarium* spp.
**T-22 G Biological Fungicide** (Rootshield Granules Biological Fungicide, T-22 G Granules Biological Fungicide)	*T. afroharzianum* (formerly *T. harzianum)*	T-22	USA[c]	agricultural field crops, fruiting vegetables, leafy vegetables, fruits, ornamental plants	*Cylindrocladium* spp., *Fusarium* spp., *Pythium* spp., *Rhizoctonia* spp., *Thielaviopsis* spp.
**T-22 Technical**	*T. afroharzianum* (formerly *T. harzianum)*	T-22	USA[c]	permitted for use on all crops	*Fusarium* spp., *Pythium* spp., *Rhizoctonia* spp., *Thielaviopsis* spp.
**T-22 WP Biological Fungicide** (Plantshield HC Biological Fungicide, Root Guardian, Rootshield AG, Rootshield AG Biological Fungicide, Rootshield Home and Garden Biological Fungicide, Rootshield Seed Treatment, Rootshield Seed Treatment Biological Fungicide, T-22 HC Biological Fungicide)	*T. afroharzianum* (formerly *T. harzianum)*	T-22	USA[c]	almonds, bananas, beans, berries, broccoli, brussels, canola, carrots, cauliflower, corn, cotton, cucumbers, ornamental plants, citrus, grapes, wheat, leafy vegetables,	*Botrytis* spp., *Fusarium* spp., *Pythium* spp., *Rhizoctonia* spp., *Thielaviopsis* spp.
**T34 Biocontrol** (Asperello T34 Biocontrol)	*T. asperellum*	T34	USA[c], EU Members[e]	carnation plants growing in the greenhouse	*F. oxysporum* f.sp. *dianthi*
**Tanus**	*T. asperellum* *T. harzianum*	URM 8120 URM 8119	Brazil[b]	permitted for use on all crops	*Rhizoctonia* spp., anthracnose disease
**Trianum DS**	*T. afroharzianum* (formerly *T. harzianum)*	T-22	Brazil[b]	permitted for use on all crops	*Macrophomina phaseolina*, *Sclerotinia sclerotiorum*, *Fusarium oxysporum* f.sp. *lycopersici*

Table S1. Continuation.

Name of products	Species	Strain	Registered in	Protected crops	Target pathogens
**Trianum G Biological Fungicide** (Trianum G, Trianum Granules Biological Fungicide, T-22 G Granules – Biological Fungicide)	*T. afroharzianum* (formerly *T. harzianum)*	T-22	USA[c]	permitted for use on all crops	*Cylindrocladium* spp., *Fusarium* spp., *Pythium* spp., *Rhizoctonia* spp., *Thielaviopsis* spp.
**Trianum Technical**	*T. afroharzianum* (formerly *T. harzianum)*	T-22	USA[c]	permitted for use on all crops	*Rhizoctonia solani*, *Fusarium* spp., *Pythium* spp.
**Trianum WG Biological Fungicide** (Trianum P Biological Fungicide)	*T. afroharzianum* (formerly *T. harzianum)*	T-22	USA[c], Brazil[b]	permitted for use on all crops	*Fusarium* spp., *Rhizoctonia* spp., *Pythium* spp., *Thielaviopsis* spp.
**Trianum P**	*T. atrobrunneum*	ITEM 908	EU Members[e]	tomato	*Pythium* spp., *Rhizoctonia* spp., *Fusarium* spp.
**Tricho Plus Biofungicide**	*T. asperelloides*	JM41R	USA[c]	greenhouse plant beds, irrigation systems	*Fusarium* spp., *Phytophthora* spp., *Pythium* spp., *Rhizoctonia* spp., *Sclerotinia* spp.
**Trichoderma harzianum WP**	*T. harzianum*		Chile[d]	permitted for use on all crops	*Botrytis cinerea*, *Phytophthora* spp.
**Trichodermax EC**	*T. asperellum*	T-211	Brazil[b]	soybean, beans	*Fusarium solani* f.sp. *glycines*, *Rhizoctonia solani*, *Sclerotinia sclerotiorum*
**Trichodermil SC 1306**	*T. harzianum*	ESALQ-1306	Brazil[b]	beans, strawberry, soybean	*Fusarium solani* f.sp. *phaseoli*, *Rhizoctonia solani*, *Sclerotinia sclerotiorum*
**Trichodermil Super SC 1306**	*T. harzianum*	ESALQ-1306	Brazil[b]	beans, strawberry, soybean	*Fusarium solani* f.sp. *phaseoli*, *Rhizoctonia solani*, *Sclerotinia sclerotiorum*
**Tricho-Guard**	*T. asperellum*	BV-10	Brazil[b]	permitted for use on all crops	*Rhizoctonia* spp.
**TrichoPlus®**	*T. fertile*	JM4 1R	Africa[j]	permitted for use on all crops	*Rhizoctonia solani*, *Pythium* spp., *Sclerotinia* spp., *Fusarium* spp., *Phytophthora* spp.

Table S1. Continuation.

Name of products	Species	Strain	Registered in	Protected crops	Target pathogens
**Trichosoil**	*T. harzianum*		Uruguay[k]	garlic, pepper, lettuce, tomato, clove, roses	*Rhizoctonia* spp., *Pythium* spp., *Botrytis* spp.
**Tricho-Turbo**	*T. asperellum*	BV-10	Brazil[b]	permitted for use on all crops	*Rhizoctonia* spp.
**Tric-O**	*T. harzianum*		Chile[d]	permitted for use on all crops	*Alternaria* spp., *Botrytis cinerea*, *Cladosporium* spp., *Fusarium* spp., *Geotrichum candidum*, *Venturia* spp.
**Tricovab**	*T. stromaticum*		Brazil[b]	cocoa trees	*Moniliophthora perniciosa*
**Tri-Soil**	*T. atroviride*	I-1237	France[l]	carrot, lettuce, potato, ornamentals	*Pythium* sp., *Rhizoctonia solani*
**Tritter**	*T. harzianum*		Brazil[b]	permitted for use on all crops	*Rhizoctonia solani*, *Sclerotinia sclerotiorum*
**Trychonyd FR25**	*T. harzianum*	CCT 6550	Brazil[b]	permitted for use on all crops	*Sclerotinia sclerotiorum*
**Tusal**	*T. asperellum*	T25	EU Members[e]	tomato, pepper, cucumber, courgetti	*Phytophthora* spp., *Fusarium* spp., *Rhizoctonia solani*, *Pythium* spp., *Sclerotinia sclerotiorum*
**Unite**	*T. atroviride*		New Zealand[g]	organic and conventional crops, orchards, vineyards, cropping and outdoor vegetables, woody trees	*Pythium* spp., *Rhizoctonia* spp., *Phytophthora* spp., *Cylindrocarpon* spp.
**Vinevax Bio-dowel**	*T. atroviride*	5 strains	New Zealand[g], Australia[h]	grapevines, fruit trees	*Eutypa lata*, *Botryosphaeria stevensii*, *Chondrostereum purpureum*
**Vinevax Pruning Wound Dressing**	*T. atroviride*	5 strains	New Zealand[g], Australia[h]	pip and stone fruit trees, ornamental trees, shrubs, grapevines	*Eutypa lata*, *Botryosphaeria* spp., *Phaeomoniella chlamydospora*
**Vintec**	*T. atroviride*	SC1	USA[c], EU Members[e]	grapevines (ornamental, nursery), almonds (dormant application)	*Phaeomoniella chlamydospora*, *Phaeoacremonium aleophylum*, *Diplodia seriata*, *Eutypa lata*, *Eutypa armenicae*, *Botryosphaeria ribis*
**Virisan**	*T. asperellum*	TV1	EU Members[e]	tomato (greenhouse, professional)	*Pythium* spp., *Rhizoctonia* spp., *Phytophthora* spp., *Phoma* spp., *Verticillium* spp., *Fusarium* spp.

Table S1. Continuation.

Name of products	Species	Strain	Registered in	Protected crops	Target pathogens
**Walker**	*T. afroharzianum* (formerly *T. harzianum*)	T-22	Brazil[b]	permitted for use on all crops	*F. oxysporum* f.sp. *lycopersici*, *Sclerotinia sclerotiorum*
**Xilon WP**	*T. asperellum*	T34	Poland[m]	tomato, pepper, cloves, ornamentals	*F. oxysporum* f.sp. *dianthi*, *Pythium* sp., *Dianthus* sp.

[a] These biological products have been registered for use in India by Central Insecticide Board and Registration Committee of the Government of India (Available online: http://ppqs.gov.in/divisions/cib-rc/bio-pesticide-registrant; https://bioprotectionportal.com/india; accessed on 24 January 2022)
[b] These biological products have been registered for use in Brazil by the Ministério da Agricultura, Pecuária e Abastecimento (Available online: https://www.gov.br/agricultura/pt-br/; https://bioprotectionportal.com/brazil; accessed on 24 January 2022)
[c] These biological products have been registered for use in USA by the United States Environmental Protection Agency (EPA) (Available online: https://www.epa.gov/ingredients-used-pesticide-products/biopesticide-active-ingredients; accessed on 24 January 2022)
[d] These biological products have been registered for use in Chile by Servicio Agrícola y Ganadero (Available online: https://www.sag.gob.cl/; https://bioprotectionportal.com/chile; accessed on 24 January 2022)
[e] These biological products have been registered for use in Member States of the European Union by the European Commission (Available online: https://ec.europa.eu/food/plants/pesticides/eu-pesticides-database_en; accessed on 24 January 2022)
[f] These biological products have been registered for use in Canada by the Canadian Food and Inspection Agency (CFIA) or Health Canada (Available online: https://inspection.canada.ca/eng/1297964599443/1297965645317/; https://www.canada.ca/en/health-canada.html/; accessed on 24 January 2022)
[g] These biological products have been registered for use in New Zealand by the Ministry for Primary Industries (Available online: https://eatsafe.nzfsa.govt.nz/web/public/acvm-register; http://agrimm.co.nz/new-zealand/; accessed on 24 January 2022)
[h] These biological products have been registered for use in Australia by the New South Wales Environment Protection Authority (EPA) (Available online: https://www.epa.nsw.gov.au/; https://services.apvma.gov.au/Pubcris; http://agrimm.co.nz/australia/; accessed on 24 January 2022)
[i] This biological product has been registered for use in Uganda by the Ministry of Agriculture, Animal Industry and Fisheries (MAAIF) of Uganda (Available online: https://www.agriculture.go.ug/; https://realipm.com/products/mazao-sustain/; accessed on 24 January 2022)
[j] This biological product has been registered for use in Africa by the Department of Agriculture, Land Reform and Rural Development (DALRRD) (Available online: https://www.agro.basf.co.za/en/Products/Overview/Fungicide/TrichoPlus.html; accessed on 24 January 2022)
[k] This biological product has been registered for use in Uruguay (Available online: https://www.lageycia.com/en/producto.php/32; accessed on 24 January 2022)
[l] This biological product has been registered for use in France (Available online: https://ephy.anses.fr/ppp/tri-soil; accessed on 24 January 2022)
[m] This biological product has been registered for use in Poland by the Ministry of Agriculture and Rural Development (Available online: https://www.gov.pl/web/rolnictwo/wyszukiwarka-srodkow-ochrony-roslin---zastosowanie; accessed on 24 January 2022)
